# Revisiting the adhesion mechanism of mussel-inspired chemistry†

**DOI:** 10.1039/d1sc05512g

**Published:** 2022-01-14

**Authors:** Chao Zhang, Li Xiang, Jiawen Zhang, Chang Liu, Zuankai Wang, Hongbo Zeng, Zhi-Kang Xu

**Affiliations:** Department of Polymer Science and Engineering, Zhejiang University Hangzhou 310027 China xuzk@zju.edu.cn; Department of Chemical and Materials Engineering, University of Alberta Edmonton Alberta T6G 1H9 Canada hongbo.zeng@ualberta.ca; Department of Mechanical Engineering, City University of Hong Kong Hong Kong 999077 China zuanwang@cityu.edu.hk; Jiangsu Key Laboratory of Construction Materials, School of Materials Science and Engineering, Southeast University Nanjing 211189 China

## Abstract

Mussel-inspired chemistry has become an ideal platform to engineer a myriad of functional materials, but fully understanding the underlying adhesion mechanism is still missing. Particularly, one of the most pivotal questions is whether catechol still plays a dominant role in molecular-scale adhesion like that in mussel adhesive proteins. Herein, for the first time, we reveal an unexplored adhesion mechanism of mussel-inspired chemistry that is strongly dictated by 5,6-dihydroxyindole (DHI) moieties, amending the conventional viewpoint of catechol-dominated adhesion. We demonstrate that polydopamine (PDA) delivers an unprecedented adhesion of 71.62 mN m^−1^, which surpasses that of many mussel-inspired derivatives and is even 121-fold higher than that of polycatechol. Such a robust adhesion mainly stems from a high yield of DHI moieties through a delicate synergy of leading oxidation and subsidiary cyclization within self-polymerization, allowing for governing mussel-inspired adhesion by the substituent chemistry and self-polymerization manner. The adhesion mechanisms revealed in this work offer a useful paradigm for the exploitation of functional mussel-inspired materials.

## Abstract 2

A 5,6-dihydroxyindole (DHI)-dominated mechanism in the interfacial adhesion of mussel-inspired chemistry is first proposed and demonstrated by the fusion of *in situ* force measurements with molecular-scale simulations.

## Graphical abstract

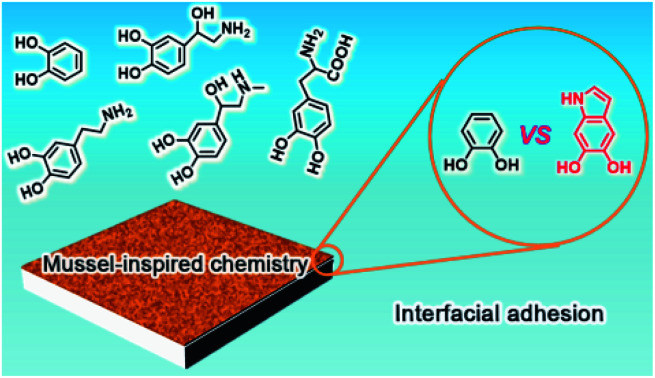

## Introduction

In nature, marine mussels have orchestrated extraordinary principles for achieving robust wet adhesion by the elegant manipulation of the secreted adhesive proteins containing 3,4-dihydroxy-l-phenylalanine (DOPA) groups.^1–3^ Since this seminal work, mussel-inspired chemistry has evolved as a powerful platform for surface engineering in many fields as diverse as bioengineering,^4–6^ energy storage and conversion,^7,8^ environmental remediation,^9^ and wearable electronics.^10–12^ Distinct from conventional methods, mussel-inspired chemistry possesses a set of intriguing merits such as surface-adaptive adhesion, ease of implementation, eco-friendly processes, and versatile function integration.^13–16^ To date, exciting achievements have been made in the synthesis and application of mussel-inspired derivatives, including dopamine, levodopa (l-DOPA), norepinephrine, polyphenols, and catechol-based polymers.^13,17–20^ In striking contrast, the fundamental mechanism, especially responsible for wet adhesion in mussel-inspired chemistry, has not been well understood so far.

Several advanced force measurement techniques have been recently deployed to investigate the adhesion mechanism of mussel-inspired chemistry, which include those involving single-molecule atomic force microscopy (SM-AFM),^21–23^ colloidal probe AFM,^24^ and surface forces apparatus (SFA).^2,25–27^ The measured results have manifested the key significance of catechol groups in forming multiple non-covalent and covalent interactions with various surfaces for contributing to the adhesion. Other moieties such as amine and phosphate ester can also work with catechol groups to synergistically enhance wet adhesion through some unique mechanisms such as cation–π interactions,^25,28^ surface salt displacement,^29,30^ and anion–π interactions.^20^ However, in spite of extensive achievements in molecular-level measurements, most cases revolve around some specific-design research objects such as single catechol molecule and mussel adhesive proteins. The catechol-dominated role in these previous research objects still remains challenging to elucidate the adhesion mechanism of mussel-inspired chemistry from an integrated perspective, because most mussel-inspired derivatives encounter a sophisticated oxidative-polymerization process to produce complex, dispersed chemical compositions.^31–33^ Taking dopamine as a typical example, its polymerization process normally involves oxidation, intramolecular cyclization, intramolecular rearrangement, and covalent coupling.^14^ As a result, the as-polymerized product, termed polydopamine (PDA), features extremely complex compositions containing catechol, leucodopaminechrome, dopaminechrome, 5,6-dihydroxyindole (DHI), 5,6-indolequinone, amine, and imine groups. Moreover, many other common mussel-inspired derivatives also bear some distinct substitutional groups such as hydroxyl, methyl, and carboxyl moieties. All of these tiny groups actually have a substantial impact on mussel-inspired chemistry from self-polymerization to eventual adhesion.^34–36^ However, to date, very few reports focus on this molecular-level impact and current views still follow the previous catechol-dominated adhesion mechanism. To this end, it is imperative to revisit mussel-inspired chemistry and uncover the underneath adhesion mechanism thoroughly from the perspectives of molecular architecture.

Herein, in striking contrast to the conventional catechol-based adhesion, we report a DHI-dominated mechanism in the interfacial adhesion of mussel-inspired chemistry from the perspectives of molecular architecture. *In situ* SFA measurements show that PDA exhibits an unparalleled adhesion as high as ∼71.62 mN m^−1^, which is approximately 121, 68, 18, and 5-fold higher than that of polycatechol, poly(l-DOPA), polyadrenaline, and polynoradrenaline, respectively. By combining the results of force measurements with molecular-scale simulations, we attribute the strong adhesion of PDA to its high yield of DHI moieties by virtue of the synergy effect of oxidation and intramolecular cyclization for forming sufficient DHI-enabled interactions (especially cation–π interaction). We also demonstrate that the substituent chemistry and polymerization manner can govern the adhesion of mussel-inspired chemistry by modulating the yield and properties of DHI moieties. Our findings offer new insights into the fundamental understanding of mussel-inspired adhesion as well as the rational design of mussel-inspired materials.

## Results and discussion

### *In situ* characterization of adhesion strength

**Fig. 1:**
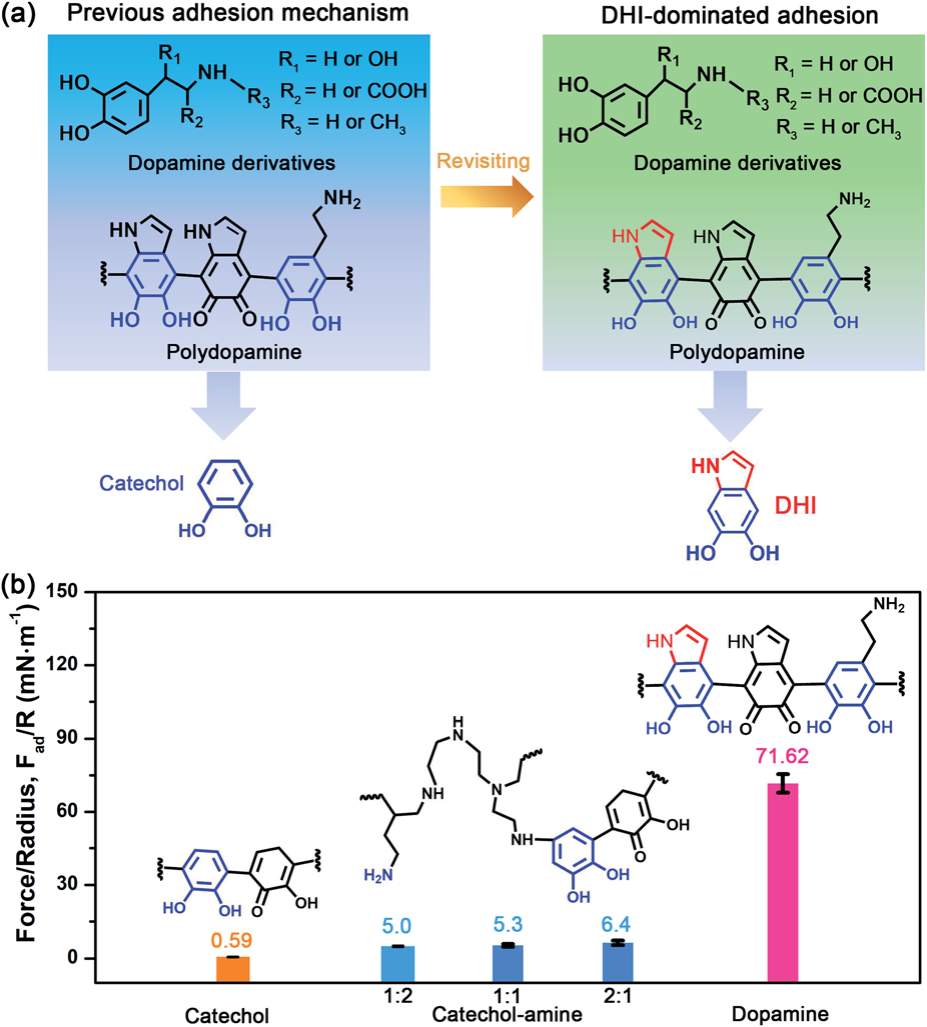
(a) Schematic illustration of the molecular architecture and polymerization product of mussel-inspired derivatives, as well as the comparison of interfacial adhesion sites between previous reports and this work. Here, mussel-inspired derivatives contain dopamine (R_1_, R_2_, R_3_ = H), l-DOPA (R_2_ = COOH; R_1_, R_3_ = H), adrenaline (R_1_ = OH; R_2_ = H; R_3_ = CH_3_), and noradrenaline (R_1_ = OH; R_2_, R_3_ = H). The molecular structure of the polymerization product is typically represented by PDA. (b) Comparison of the adhesion strength of catechol, catechol–amine (PEI 800), and dopamine after 1 h *in situ* polymerization. The molar ratio of catechol and amine (PEI 800) was tuned from 1 : 2 to 1 : 1 and 2 : 1. Note that catechol and dopamine have the same molar concentration. The inset images show the molecular structures of polycatechol, poly(catechol-amine), and PDA, respectively. The results show that the adhesion of PDA is several times higher than that of catechol groups and their synergy with amine groups, demonstrating that DHI moieties play a dominant role in adhesion over the conventional catechol group.

To revisit the adhesion mechanism of mussel-inspired chemistry and investigate the impact of molecular architecture and the polymerization manner, five kinds of mussel-inspired derivatives were selected as model objects, including catechol, dopamine (R_1_, R_2_, R_3_ = H), l-DOPA (R_2_ = COOH; R_1_, R_3_ = H), adrenaline (R_1_ = OH; R_2_ = H; R_3_ = CH_3_), and noradrenaline (R_1_ = OH; R_2_, R_3_ = H) (Fig. 1a), which possess a similar ability to self-polymerize into catechol-based adhesives though their molecular architecture and polymerization rate vary. We first monitored the adhesion evolution of dopamine during the *in situ* polymerization process at pH 8.5 in a symmetric configuration using the SFA (Fig. S1†). The adhesion of PDA has a gentle increase with the polymerization time and reaches a peak value of 71.62 mN m^−1^ (adhesion energy per unit area *W*_ad_ of ∼15.21 mJ m^−2^) after 1 h (Fig. 1b and S2†). However, this adhesion dramatically decreases to 52.82 mN m^−1^ (*W*_ad_ = 11.21 mJ m^−2^), with extending the polymerization time to 2 h. The deteriorated adhesion results from the enhanced steric repulsion between the two interacting surfaces, because of the generation of large PDA aggregates that lead to relatively higher surface roughness (*i.e.*, ∼1.54 nm, three times higher than that of 1 h deposition) (Fig. S3†). Still, the adhesion strength of PDA outperforms that of most reported mussel adhesive proteins and mussel-inspired coatings.^25,30,37–39^ Remarkably, such robust adhesion can remain almost unchangeable during consecutive force measurements at the same interaction position (Fig. S4†), suggesting that PDA-based adhesion is totally reversible and should be caused by non-covalent interactions.

Elucidating which groups are mainly responsible for the strong adhesion of PDA can be implemented by splitting dopamine into single functional moieties (*e.g.*, catechol) as controls. We first selected catechol, the simplest mussel-inspired derivative that is able to self-polymerize into polycatechol, to evaluate its contribution in PDA-based adhesion. Fig. 1b illustrates that only a weak adhesion of 0.59 mN m^−1^ (*W*_ad_ = 0.13 mJ m^−2^) is measured between two polycatechol layers, which is 121-fold lower than that of PDA. Considering that the molecular structure of polycatechol is short of amine groups compared with PDA, we further measured the adhesion of catechol and amine (*i.e.*, polyethyleneimine, PEI 800) with various molar ratios, in which catechol and amine can form poly(catechol-amine) by the Michael reaction (Fig. S5†). As shown in Fig. 1b and S6,† the adhesion of poly(catechol-amine) significantly increases compared with catechol and can be tuned from 5.0 mN m^−1^ (*W*_ad_ = 1.06 mJ m^−2^) to 6.4 mN m^−1^ (*W*_ad_ = 1.36 mJ m^−2^), yet it is still ten times lower than that of PDA. Even though catechol is replaced by polyphenols like tannic acid containing numerous catechol groups, the maximized adhesion of poly(tannic acid–amine) is only 20.86 mN m^−1^ (*W*_ad_ = 4.43 mJ m^−2^),^40^ which is still far away from that of PDA. In addition to the adhesion between coatings, we further studied the adhesion between various coatings (polycatechol, poly(catechol-amine), and PDA) and mica in an asymmetric measurement configuration by using an SFA. As shown in Fig. S7,† the adhesion between PDA and mica (12.7 mN m^−1^) is 3-fold and 15-fold higher than that of poly(catechol-amine) (4.2 mN m^−1^) and polycatechol (0.83 mN m^−1^), respectively. These adhesion results in both symmetric and asymmetric measurement configurations manifest that PDA has stronger adhesion capability than polycatechol and poly(catechol-amine). Therefore, we speculate that the strong adhesion of PDA originates from an unexplored interaction mechanism, which is different from the conventional PDA-based adhesion and mussel adhesive proteins that rely on the major contribution of catechol groups and their synergy with amine groups.^28,41^

### DHI-dominated adhesion mechanism

**Fig. 2:**
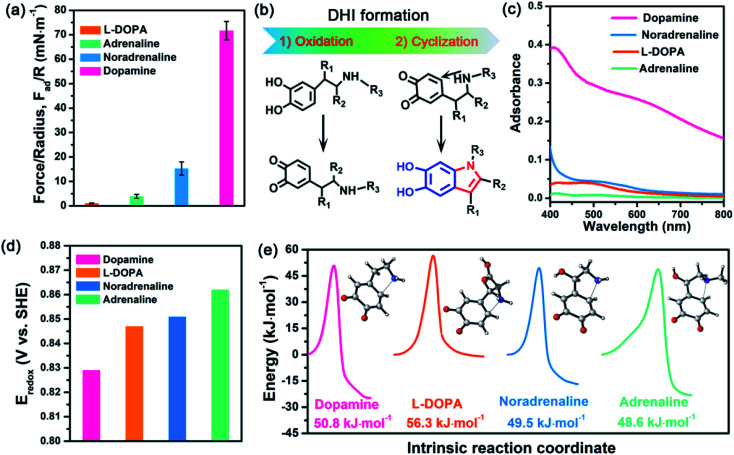
(a) Adhesion of l-DOPA (R_2_ = COOH; R_1_, R_3_ = H), adrenaline (R_1_ = OH; R_2_ = H; R_3_ = CH_3_), noradrenaline (R_1_ = OH; R_2_, R_3_ = H), and dopamine (R_1_, R_2_, R_3_ = H) after *in situ* polymerization at pH 8.5 for 1 h. (b) Schematic illustration of the major process of DHI formation, in which the substituents can alter the chemical micro-environment of oxidation and cyclization sites. (c) UV-vis spectra of l-DOPA, adrenaline, noradrenaline, and dopamine at pH 8.5 after 1 h polymerization. (d) Standard electrode potentials of l-DOPA, adrenaline, noradrenaline, and dopamine during the oxidation process at pH 8.5. (e) Energy barriers of the oxidized l-DOPA, adrenaline, noradrenaline, and dopamine during intramolecular cyclization. The inset images show the transition state of different mussel-inspired derivatives in the Michael addition.

Further inspecting the molecular architecture of PDA, polycatechol, and poly(catechol-amine) (inset images in Fig. 1b), we find that PDA comprises a large number of indolic moieties, especially 5,6-dihydroxyindole (DHI),^32,42–45^ due to the presence of intramolecular cyclization which is not achievable in the cases of catechol and catechol-amine. Moreover, DHI moieties can form much stronger interactions than the catechol group, which can be fully evidenced by our molecular-level simulations (Fig. S8 and S9†) as well as previous coating deposition results.^43,46,47^ Thus, we hypothesize that DHI moieties probably play a key role in PDA-based adhesion instead of conventional catechol-dominated adhesion (Fig. 1a). To manifest this conjecture, we further quantified the adhesion of three other dopamine derivatives, such as l-DOPA (R_2_ = COOH; R_1_, R_3_ = H), adrenaline (R_1_ = OH; R_2_ = H; R_3_ = CH_3_), and noradrenaline (R_1_ = OH; R_2_, R_3_ = H), all of which possess dopamine-like capacities to generate DHI moieties and form nanoscale-roughness coatings (Fig. S10†). The measured adhesion of poly(l-DOPA), polyadrenaline, and polynoradrenaline is 1.06 mN m^−1^ (*W*_ad_ = 0.23 mJ m^−2^), 3.89 mN m^−1^ (*W*_ad_ = 0.83 mJ m^−2^), and 15.28 mN m^−1^ (*W*_ad_ = 3.24 mJ m^−2^), respectively (Fig. 2a and S11†). As compared with polycatechol that lacks DHI, all these derivatives show several times higher adhesion strength, which also underpins the significant role of DHI in adhesion. Moreover, their adhesion strengths are 68, 18, and 5-fold lower than that of PDA, attributed to the presence of special substituents. To be more specific, l-DOPA and noradrenaline possess a carboxyl group (R_2_ = COOH) and hydroxyl group (R_1_ = OH) as the electron-withdrawing and electron-donating substituent, respectively, whereas adrenaline has a methyl substituent (R_3_ = CH_3_) to become a secondary amine compared with dopamine. These substituents are capable of governing the chemical micro-environment of oxidation and cyclization sites, determining the formation and properties of DHI moieties (Fig. 2b), and further affecting DHI-enabled interactions.

### Substituent effect on DHI formation

To study the substituent chemistry effect on DHI formation, the UV-vis spectra of l-DOPA, adrenaline, noradrenaline, and dopamine after polymerization were characterized. By analyzing the UV-vis absorbance,^48^ the forming capacities of DHI obey the sequence of dopamine > noradrenaline > l-DOPA > adrenaline (Fig. 2c). The substituent chemistry impact can be further manifested by molecular-scale simulations to study two important steps: oxidation and cyclization (Fig. 2b). By calculating the standard electrode potentials of catechol-to-quinone translation, the oxidation ability of these derivatives decreases in the sequence of dopamine (0.829 eV) > l-DOPA (0.847 eV) > noradrenaline (0.851 eV) > adrenaline (0.862 eV) (Fig. 2d). In contrast to other counterparts, dopamine exhibits the strongest oxidation ability, whereas adrenaline is the most difficult one to oxidize. This is because the substituents alter the electron loss abilities of phenolic hydroxyls and further suppress the oxidation of catechol. The intramolecular cyclization of oxidized derivatives was also investigated by calculating the energy barrier of the cyclization process. Fig. 2e indicates that the calculated energy barriers reflect the cyclization activity sequence of adrenaline (48.60 kJ mol^−1^) > noradrenaline (49.48 kJ mol^−1^) > dopamine (50.75 kJ mol^−1^) > l-DOPA (56.53 kJ mol^−1^). Remarkably, adrenaline possesses the highest cyclization activity, whereas l-DOPA exhibits the poorest cyclization activity, which are distinct from their oxidation abilities. Such a huge difference results from the substituent impact on the electron densities of the amine group. For instance, the carboxylate group of l-DOPA can pair with its cationic amine so as to impact the activity of Michael addition based cyclization.

Guided by the molecular-scale simulation results, dopamine is found to display the strongest oxidation ability yet the second poorest cyclization activity, whereas adrenaline possesses the weakest oxidation ability yet the highest cyclization activity (Fig. 2d and e). Considering that dopamine and adrenaline exhibit the highest and lowest yield of DHI by UV-vis analysis (Fig. 2c), respectively, the first-step oxidation is concluded as a rate-determining step for generating DHI moieties compared with cyclization. Comparison of l-DOPA and noradrenaline showed that although noradrenaline shows a slower oxidation activity, it has better cyclization ability than l-DOPA (Fig. 2d and e). By combining the results of noradrenaline and l-DOPA which have similar DHI yield, we conclude that cyclization is also of great importance and plays a synergistic assistance role in DHI formation. Taking all the results together, DHI moiety formation in mussel-inspired derivatives is a complex, synergistic process: the first-step oxidation plays a leading role, and the subsequent cyclization plays an assistant role. Such a highly efficient cooperation process endows dopamine with the highest yield of DHI moieties, followed by noradrenaline, l-DOPA, and adrenaline. Moreover, manipulating substituent chemistry to enhance the electron loss abilities of phenolic hydroxyls and the electron densities of amine groups is an effective approach to improve the yield of DHI moieties.

### Working mechanism of DHI on adhesion

**Fig. 3:**
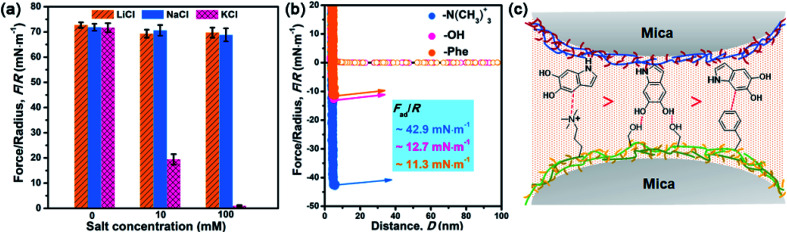
(a) Adhesion strength of dopamine with different salts (LiCl, NaCl, and KCl) after 1 h *in situ* polymerization. The salt concentration is adjusted from 0 mM to 100 mM. The results suggest that the adhesion of PDA has no obvious change at different concentrations of LiCl and NaCl yet is highly sensitive on the concentration of KCl. (b) Adhesion strength between the PDA coating and various surfaces (OH-, Phe-, and N(CH_3_)_3_^+^-terminated surface) using an asymmetric SFA measurement configuration. The results indicate that the cation–π interaction is a major contributor in PDA adhesion, whereas π–π interactions and H-bonding are also of significance and play an auxiliary role. (c) Schematic illustration of intermolecular interactions between the PDA coating and various surfaces: OH-, Phe-, and N(CH_3_)_3_^+^-terminated surface. It illustrates that the cation–π interaction is much stronger than both the π–π interaction and H-bonding in PDA adhesion.

After understanding the substituent chemistry effect on DHI formation, we try to elucidate which interactions DHI are mainly responsible for, which is very critical to figure out the adhesion mechanism of DHI. We first investigated the adhesion of *in situ* polymerized dopamine in response to salt species (*i.e.*, LiCl, NaCl, and KCl) and their concentrations. As shown in Fig. 3a and S12,† the adhesion only slightly changes with the addition of LiCl or NaCl, even upon a wide-ranging concentration from 0 mM to 100 mM. In contrast, the adhesion decreases dramatically as the concentration of KCl increases. For instance, the measured adhesion dramatically decreases from 71.62 mN m^−1^ (*W*_ad_ = 15.21 mJ m^−2^) to 19.38 mN m^−1^ (*W*_ad_ = 4.11 mJ m^−2^) with the increase of KCl concentration to 10 mM. This Li^+^/Na^+^-insensitive yet K^+^-sensitive adhesion behavior coincides well with the cation–π interaction, in which K^+^ can form stronger cation–π interactions with DHI moieties compared with Li^+^ and Na^+^.^25,49^ As a result, PDA adhesion mainly originates from DHI-enabled cation–π interactions over the other interactions such as π–π interactions, hydrogen bonds, and hydrophobic interactions because they are normally insensitive to salt species and concentrations. We further examined the contribution of these DHI-enabled interactions by measuring the adhesion of PDA to Phe-, OH-, and N(CH_3_)_3_^+^-terminated surfaces in an asymmetric configuration using the SFA. Fig. 3b shows that the adhesion between PDA and a N(CH_3_)_3_^+^-terminated surface is as high as 42.9 mN m^−1^ (cation–π interaction), which far surpasses that of Phe-terminated surface (11.3 mN m^−1^, π–π interaction or hydrophobic interaction) and OH-terminated surface (12.7 mN m^−1^, hydrogen bond) (Fig. 3c). Together, we deduced that DHI-enabled cation–π interactions play a major role in PDA adhesion, whereas DHI-enabled π–π interactions, hydrophobic interactions, and hydrogen bonding are also of significance and play an auxiliary role.

**Fig. 4:**
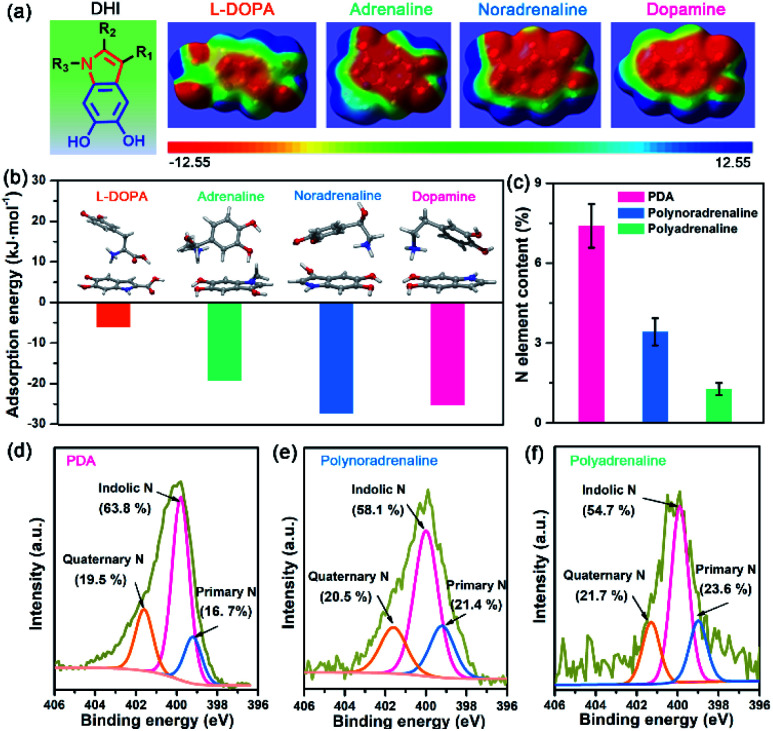
(a) Molecular structure and electrostatic potential (ESP) surfaces of DHI moieties with different substituents: l-DOPA (R_2_ = COOH; R_1_, R_3_ = H), adrenaline (R_1_ = OH; R_2_ = H; R_3_ = CH_3_), noradrenaline (R_1_ = OH; R_2_, R_3_ = H), and dopamine (R_1_, R_2_, R_3_ = H). The electrostatic potentials are mapped onto the electron density surfaces with an isovalue of 0.001 electrons per bohr^3^. The color scale ranges from −12.55 (red) to 12.55 kcal mol^−1^ (blue). (b) Optimized structures and adsorption energies between DHI moieties and cationic amines in a water environment, showing distinct cation–π interaction strengths caused by different substituents. The inset images show the optimized geometries of different mussel-inspired derivatives and the associated DHI. (c) Comparison of N element content in PDA, polynoradrenaline, and polyadrenaline coatings after 1 h deposition. Each sample was tested at least three times. (d–f) High-resolution XPS spectra of N 1s for PDA, polynoradrenaline, and polyadrenaline coatings. Specific binding energies are assigned to indolic N (399.7 eV), quaternary N (401.6 eV) and primary N (398.8 eV).

Owing to different π-conjugated electron densities within DHI caused by the substitutes (Fig. 4a), dopamine derivatives exhibit distinct capacities to form mussel-inspired interactions, especially cation–π interactions. Their cation-π interaction strength in an aqueous environment can be revealed by density functional theory (DFT) simulations. As displayed in Fig. 4b, the calculated adsorption energies of cation–π interactions are 6.04 kJ mol^−1^, 19.21 kJ mol^−1^, 27.36 kJ mol^−1^, and 25.25 kJ mol^−1^ in the case of l-DOPA, adrenaline, noradrenaline, and dopamine, respectively. In striking contrast to the others, l-DOPA exhibits the lowest cation–π interaction strength because of its electron-withdrawing substituent (R_2_ = COOH) which significantly decreases the electron densities of DHI (Fig. 4a). Such low cation–π interaction strength can be used to well explain why l-DOPA displays the poorest adhesion ability (Fig. 2a). Notably, the cation–π interaction strength of noradrenaline is the strongest, even slightly higher than that of dopamine, because of the higher electron densities of its DHI moieties stemming from the electron-donating substituent (R_1_ = OH) (Fig. 4a). Thus, in addition to impacting the yield of DHI moieties, the substituent chemistry is also able to regulate the properties of DHI (*i.e.*, π-electron density) and hence the strength of DHI-enabled cation–π interactions.

Next, we investigated the number of DHI-enabled cation–π interaction sites in the coatings of PDA and derivatives using XPS spectra. Interestingly, the N element content in PDA is around 7.40%, which is 2.2- and 5.6-fold higher than that of polynoradrenaline (3.42%) and polyadrenaline (1.27%) (Fig. 4c and Table S1†). By further analyzing their high-resolution N 1s XPS spectra, the proportion of DHI moieties (399.7 eV) in PDA is calculated to be 63.8% (Fig. 4d), as opposed to that of polynoradrenaline (58.1%) and polyadrenaline (54.7%) (Fig. 4e and f). For cationic amine moieties (401.6 eV), they have a very comparable ratio (Fig. 4d–f). In combination with N content and the ratio of moieties, the overall amount of both DHI and cationic amine moieties in PDA is demonstrated to be far higher than that of polynoradrenaline and polyadrenaline, implying that PDA can generate more DHI-enabled interaction sites. As a result, in spite of the second strongest cation–π binding strength, PDA still gives the highest adhesion strength, followed by polynoradrenaline and polyadrenaline. These findings create a blueprint that manipulating substituent chemistry to govern the yield and properties of DHI offers a new and powerful route to enhance wet adhesion of mussel-inspired materials.

### Generality of DHI-dominated adhesion

**Fig. 5:**
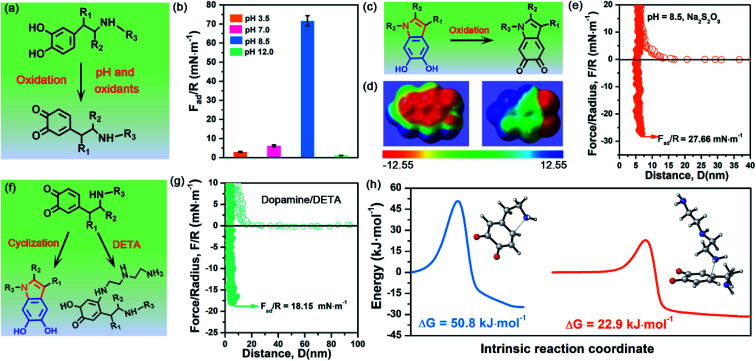
(a) Schematic illustration of the oxidation process of dopamine impacted by pH and oxidants. (b) Adhesion of dopamine after 1 h *in situ* polymerization at different pH values. (c) Molecular structure change of DHI moieties after adding Na_2_S_2_O_8_. (d) Electron density of DHI moieties before and after adding Na_2_S_2_O_8_. The electrostatic potentials are mapped onto the electron density surfaces with an isovalue of 0.001 electrons per bohr^3^. The color scale ranges from −12.55 (red) to 12.55 kcal mol^−1^ (blue). (e) Representative force–distance curves of dopamine in the presence of Na_2_S_2_O_8_. (f) Schematic illustration of a competition process to affect the cyclization of dopamine by introducing DETA. (g) Representative force–distance curves of dopamine and DETA at pH 8.5. (h) Energy barrier of Michael addition: intramolecular cyclization of dopamine (blue line), and the oxidized dopamine and DETA (yellow line). The inset images show the transition state of intramolecular cyclization of dopamine and the reaction of oxidized dopamine and DETA.

To demonstrate the validity and generality of DHI-dominated adhesion, we also examined the effect of many synthesis conditions that may vary the yield and properties of DHI and affect DHI-enabled interactions (Fig. 5a). First, we investigated the interfacial adhesion of *in situ* polymerized dopamine at a wide range of pH values from acidity to alkalinity. The measured adhesion at pH 3.5 and pH 7.0 is 2.98 mN m^−1^ (*W*_ad_ = 0.63 mJ m^−2^) and 6.18 mN m^−1^ (*W*_ad_ = 1.31 mJ m^−2^), respectively, which are extremely weaker than that at pH 8.5 (*i.e.*, 71.62 mN m^−1^) (Fig. 5b and S13†). Such a huge difference arises from their distinct yields of DHI during the polymerization process. In weak acidic and neutral media, the first-step oxidation kinetics of dopamine is greatly suppressed,^50,51^ and the amine groups tend to be protonated and deteriorate the intramolecular cyclization, thus giving rise to low throughput of DHI.^52^ Such low DHI yield inevitably decreases the amount of potential interaction sites and eventually gives weak adhesion. Further increasing the pH value from 8.5 to 12, the adhesion dramatically decreases to a mediocre value of 1.11 mN m^−1^ (*W*_ad_ = 0.24 mJ m^−2^), which highly coincides with very limited deposition capability of dopamine under strong alkaline conditions (*i.e.*, pH > 10).^53,54^ In strong alkaline media, despite possessing high yield of DHI moieties, the resultant DHI moieties are fast, substantially oxidized into 5,6-indolequinone because of the large dissociations of phenolic hydroxyl groups, which destroys the π-conjugated electron densities within DHI and thus weakens DHI-enabled interactions including cation–π interactions, π–π interactions and hydrogen bonding (Fig. 5c and d). Second, considering that many oxidants show superior ability to enhance the polymerization and deposition of mussel-inspired derivatives,^55,56^ we also studied the influence of oxidants on the interfacial adhesion of PDA. Fig. 5e demonstrates that, after the incorporation of oxidants (*i.e.*, Na_2_S_2_O_8_), the measured adhesion has an obvious decrease from 71.62 mN m^−1^ (*W*_ad_ = 15.21 mJ m^−2^) to 27.66 mN m^−1^ (*W*_ad_ = 5.87 mJ m^−2^). This is because Na_2_S_2_O_8_, a stronger oxidant compared with oxygen, can induce more oxidation of DHI into 5,6-indolequinone, which undermines DHI-enabled interactions and eventually results in a reduced adhesion.

The DHI-dominated adhesion mechanism can be further demonstrated by inspecting the adhesion variation of PDA after incorporating amine-terminated molecules. We selected diethylenetriamine (DETA) as a typical amine molecule to carry out a similar SFA experiment (Fig. 5f). Fig. 5g shows that the adhesion of PDA/DETA is as low as 18.15 mN m^−1^ (*W*_ad_ = 3.85 mJ m^−2^), which is 4-fold smaller than that of then pure PDA case. It suggests that the introduction of cationic amine significantly deteriorates PDA adhesion. Such a dramatically decreased adhesion results from the reduction of DHI yield by a competition mechanism, in which DETA can react with the oxidized dopamine by Michael addition for undermining the cyclization of dopamine (Fig. 5f). To demonstrate this competition mechanism, we further calculated the energy barrier of the Michael addition reaction between DETA and oxidized dopamine. As illustrated in Fig. 5h, their Michael addition reaction shows a much lower energy barrier of 22.9 kJ mol^−1^, as opposed to the cyclization of dopamine (50.75 kJ mol^−1^), indicating that DETA is more prone to react with the oxidized dopamine. As a result, the yield of DHI obtained by cyclization suffers from an obvious decrease, eventually leading to an obvious reduction of DHI-enabled interactions and adhesion. Together, these clear facts powerfully underpin the reliability of DHI-dominated adhesion over both catechol and amines in mussel-inspired chemistry, as well as offer a general, viable strategy to dynamically regulate mussel-inspired adhesion by the meticulous control of oxidation and cyclization.

## Conclusions

In summary, we discover and demonstrate the dominant role of DHI moieties in the interfacial adhesion of mussel-inspired chemistry over the conventional catechol group from the perspectives of molecular architecture and polymerization mechanisms. The SFA measurement results show that the adhesion of PDA is as high as 71.62 mN m^−1^, which is 121, 68, 18, and 5-fold higher than that of polycatechol, poly(l-DOPA), polyadrenaline and polynoradrenaline, respectively. Such robust adhesion probably stems from the highly efficient yield of DHI by the synergy of oxidation playing a leading role and cyclization an assistant role. Moreover, the DHI-dominated adhesion can be elegantly governed by manipulating the substituent chemistry and polymerization manner to tailor the formation and properties of DHI. From a broader perspective, our work provides a new paradigm for re-understanding fundamental adhesion science of mussel-inspired chemistry, as well as re-thinking the design principle of developing advanced mussel-inspired materials.

## Data availability

All data needed to evaluate the conclusions in the paper are present in the paper and/or ESI.†

## Author contributions

Z.-K. X., H. Z., and C. Z. conceived the research. C. Z. and L. X. designed the experiment and prepared the samples. J. Z. and C. L. conducted the molecular simulations. Z.-K. X., H. Z., Z. W., and C. Z. wrote the paper, and all the authors discussed the manuscript.

## Conflicts of interest

There are no conflicts to declare.

## Supplementary Material

SC-013-D1SC05512G-s001
