# Supplementary material for: Revisiting the adhesion mechanism of mussel-inspired chemistry
Source: Chem Sci. 2022 Jan 14;13(6):1698–705. doi: 10.1039/d1sc05512g (PMC8827048; doi:10.1039/d1sc05512g)
Supplement: SC-013-D1SC05512G-s001 [file SC-013-D1SC05512G-s001.pdf]

Electronic Supplementary Material (ESI) for Chemical Science.
This journal is © The Royal Society of Chemistry 2022

Supplementary Information

Revisiting the adhesion mechanism of mussel-inspired chemistry

Chao Zhang[a,b†], Li Xiang[b†], Jiawen Zhang[b†], Chang Liu [b†], Zuankai Wang[c*], Hongbo Zeng[b*], and Zhi-Kang Xu[a*]

[a]Department of Polymer Science and Engineering, Zhejiang University, Hangzhou 310027, China. E-mail: xuzk@zju.edu.cn (Z.-K.X.)

[b]Department of Chemical and Materials Engineering, University of Alberta, Edmonton, Alberta, T6G 1H9 (Canada). E-mail: hongbo.zeng@ualberta.ca (H.Z.)

[c]Department of Mechanical Engineering, City University of Hong Kong, Hong Kong 999077, China. E-mail: zuanwang@cityu.edu.hk (Z.W.)

[d]Jiangsu Key Laboratory of Construction Materials, School of Materials Science and Engineering, Southeast University, Nanjing 211189, China.

[†]The four authors contributed equally to this work.

Experimental section

Materials. Dopamine hydrochloride, catechol, polyethyleneimine (PEI, M_w = 800 g/mol), diethylenetriamine (DETA), L-DOPA, adrenaline, noradrenaline, iodomethane (CH_3I), dichloromethane, 2-phenylethylmercaptan, 2-mercaptoethanol, and 2-dimethylaminoethanethiolhydrochloride were obtained from Sigma-Aldrich (Canada). Potassium chloride (KCl), ethanol, methanol, lithium chloride (LiCl), sodium chloride (NaCl), sodium persulfate ($Na_2S_2O_8$), hydrochloric acid (HCl), tris-(hydroxymethyl)aminomethane, and sodium hydroxide (NaOH) were purchased from Fisher Scientific (Canada). All chemical reagents were used without further purification. Deionized water (18.2 MΩ·cm) was used in all experiments.

Fabrication and characterization of mussel-inspired coatings. Taking dopamine as a typical example, dopamine hydrochloride (5 mM) was first dissolved in Tris buffer solution (pH = 8.5, 50 mM), followed by the immersion of fresh mica into the deposition precursor for 1 h at 25 °C. Subsequently, the as-formed PDA-coated mica was washed by deionized water and dried with nitrogen gas before use. All other mussel-inspired derivatives were carried out the similar deposition procedures. Notably, the deposition conditions of these dopamine derivatives will be optimized to guarantee the formed coatings with small root-mean-square (rms) surface roughness, which is beneficial for eliminating the interference of surface roughness on force measurement. The surface morphologies and chemical components of mussel-inspired coatings were characterized by atomic force microscope (MFP-3D, Asylum Research, Santa Barbara, CA) and X-ray photoelectron spectroscopy (XPS, PerkinElmer, USA), respectively. Additionally, to study the DHI moieties formation of mussel-inspired derivatives, an ultraviolet spectro-photometer (UV 2450, Shimadzu, Japan) was employed to meausre their UV-vis absorption spectra from 400 nm to 800 nm.

Adhesion measurement in a symmetric configuration using SFA. The normalized force (F/R)-distance (D) profile of mussel-inspired derivatives was in situ measured by SFA according to the previous reports.[1-3] In a typical symmetric force measurement, back-silvered thin mica sheets (1-5 μm) were glued onto cylindrical silica disks (R = 2 cm) by an epoxy glue and then mounted into SFA chamber in a cross-cylinder configuration. After that, a droplet of 70 μL freshly prepared mussel-inspired derivatives Tris buffer solution was injected between two mica surfaces and the system was allowed to equilibrate for 30 min. The normal forces F between in situ polymerized coatings were detected as a function of surface separation D with the distance accuracy down to 0.1 nm. The measured normal force or "pull-off" force F_{ad} was correlated to the adhesion energy per unit area between two flat surfaces W_{ad} by $F_{ad}/R = 1.5\pi W_{ad}$, dictated by the Johnson-Kendall-Roberts (JKR) model.[4] Note that, the reference distance (D = 0) was determined at the contact between two bare mica surfaces in air before the introduction of the mussel-inspired precursor. To study the synergy of catechol and amine on the impact of PDA adhesion, we measured the adhesion of catechol and PEI 800 with various molar ratios from 1:2 to 1:1 and 2:1 as controls. For obtaining repeatable results, the force measurements were conducted on at least two pairs of surfaces independently prepared with three different positions on each pair of the surfaces under the same experimental conditions.

Adhesion measurement in an asymmetric configuration using SFA. To measure the adhesion between PDA and different surfaces, we first fabricated Phe-, OH-, and $N(CH_3)_3^+$-terminated surfaces by functionalizing the gold-coated micas. Briefly, freshly gold-coated micas were immersed in 10 mM methanol solutions of phenylethyl mercaptan, 2-Mercaptoethanol, 2-(dimethylamino)ethanethiolhydrochloride for 24 h incubation. For the $N(CH_3)_3^+$-terminated surface, the as-formed 2-(dimethylamino)ethanethiolhydrochloride surface was required to carry

out the quaternization treatment of the amine groups by the immersion into dichloromethane of CH_3I. Finally, the functionalized micas were washed by ethanol and dried with nitrogen gas before use.

In a typical asymmetric force measurement, PDA-coated and functionalized mica sheets (1-5 μm) were glued onto cylindrical silica disks (R = 2 cm) by an epoxy glue and then mounted into SFA chamber in a cross-cylinder configuration. After that, a droplet of 70 μL freshly Tris buffer solution was injected between two mica surfaces and the system was allowed to equilibrate for 30 min. The normal forces F between PDA and Phe-, OH-, and $N(CH_3)_3^+$-terminated surfaces were detected as a function of surface separation D with the distance accuracy down to 0.1 nm.

Simulation calculation of the standard electrode potentials of catechol-to-quinone translation and the energy barrier of Michael addition. Geometry optimizations of all molecules were performed by density functional theory (DFT) at the M06-2X level of theory[5] with 6-311+G(d) basis set[6]. Single point energies were calculated at the M06-2X/6-31+G(d) level of theory including Grimme's D3 (zero-damping) dispersion corrections.[7] In particular, when calculating the oxidation-reduction potential, the gaseous ground state single point energies of all molecules were calculated by the thermodynamic combination method CBS-QB3.[8] Energy minima and transition states were verified through vibrational analysis.[9] All minima were found to have no imaginary frequency, while all transition states had a single imaginary frequency. The associated eigenvectors were confirmed to correspond to the motion along the reaction coordinate using the intrinsic reaction coordinate (IRC) method.[10] All calculations in aqueous solutions were used the SMD model of the self-consistent reaction field (SCRF) to describe the influence of the solvent.[11] All calculations were conducted with the Gaussian 16 software package. [12] Optimized structures were illustrated using CYLview. [13]

The redox potential of dopamine and its derivatives can be calculated from Nerst equation by the Gibbs free energy change in the aqueous solution ($\Delta G_{(sol)}$) in the following thermodynamic cycle.

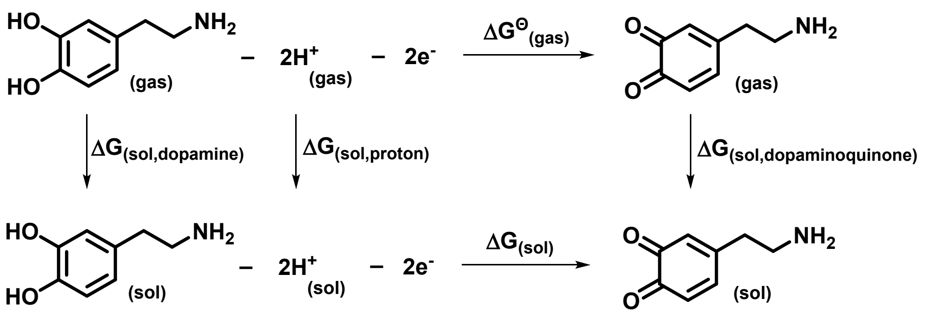

Where the Gibbs free energy changes at each step ($\Delta G_{(sol,i)}$) were obtained by DFT calculations. The oxidation-reduction potential relative to the standard hydrogen electrode (SHE) can be calculated by the Nernst equation:

$$\Delta G_{(sol)} = \Delta G_{(sol,dopaminoquinone)} - \Delta G_{(sol,dopamine)} + \Delta G_{(sol,proton)}$$

$$E^0_{redox} = -\frac{\Delta G_{(sol)}}{nF} - 4.44$$

Where n is the number of transferred electrons and F is the Faraday constant (F= 23.061 kcal mol^{-1}).

Molecular-scale simulations of the electron density and adsorption energies of cation-π interactions. To reveal the electron density of the cyclized mussel-inspired derivatives, the density functional theory (DFT) calculations were performed using Gaussian 09 software. The electrostatic potential (ESP) surfaces of cyclized L-DOPA, adrenaline, noradrenaline, and dopamine were computed and mapped onto the electron density surfaces with isovalue of 0.001 electrons/Bohr^3. The interactions within the cyclized moieties have been simulated at b3lyp/6-31g* level. The adsorption energies in water were obtained by single-point energy calculation based on configurations optimized in vacuum with zero-point vibrational correction employing polarizable continuum model (PCM).

Calculation of pair interaction energy between DHI/catechol and different groups

Molclus[14] was used to randomly generate 30 molecule pairs with different relative positions, and xtb[15] was invoked to roughly screen the lowest-energy configurations at GFN1-xTB level of theory[16] with GBSA water model. Geometry optimizations and single point energies of all lowest-energy configurations were performed by density functional theory (DFT) at the B3LYP level of theory[5] with 6-311+G(d,p) basis set[17] including Grimme's D3 (BJ-damping) dispersion corrections[18] using Gaussian 16 software package[12]. All DFT calculations were used the SMD model of the self-consistent reaction field (SCRF) to describe the aqueous environment[11]. Pair interaction energies were calculated as binding energy of each lowest-energy configuration.

Statistical Analysis. To evaluate the data reliability, three pairs of independently prepared mica surfaces were utilized to conduct the force measurements. Adhesion was measured between two as-formed coatings after in situ polymerization for a certain time. The measured adhesion was presented as the mean ± standard deviation, in which the mean value could be obtained through the equation of $\bar{F}_{ad} = \sum_{i=1}^{3} F_{adi} \Big/ n$, and the standard deviation could be determined through the equation of $S = \sqrt{\sum_{i=1}^{3} (F_{adi} - F_{ad})^2 \Big/ n}$, where n is the number of force measurements.

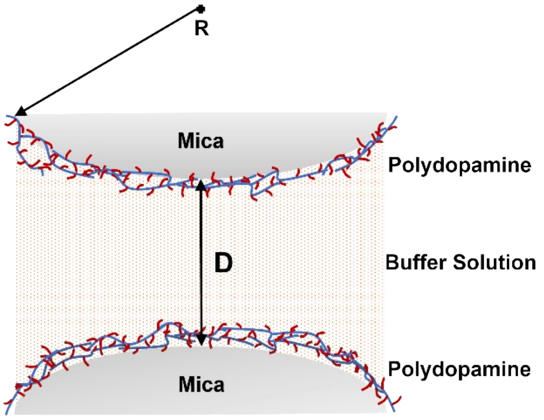

Figure S1. Schematic illustration of the adhesion measurement of dopamine and its derivatives after in situ polymerization at pH 8.5 using a symmetric SFA measurement configuration.

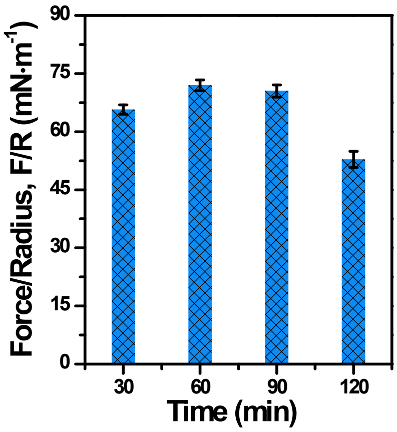

Figure S2. Time-dependent adhesion of PDA on the mica after in situ polymerization under pH 8.5: 0.5 h, 1 h, 1.5 h and 2 h.

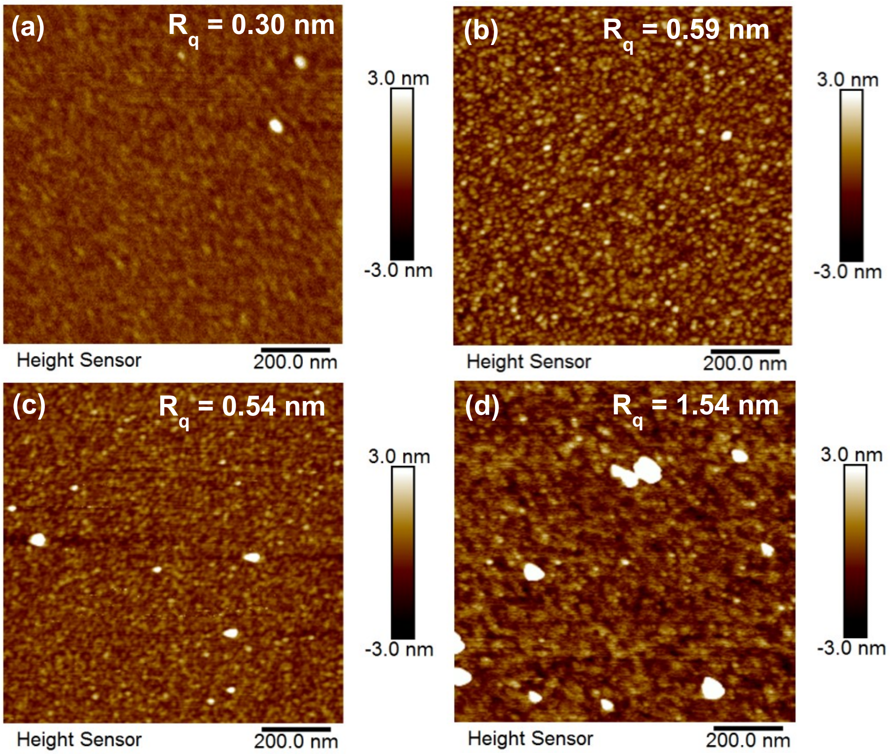

Figure S3. AFM images of PDA coatings on the mica after different deposition times under pH 8.5: (a) 0.5 h, (b) 1 h, (c) 1.5 h, and (d) 2 h. Here, dopamine concentration is fixed to be 5 mM.

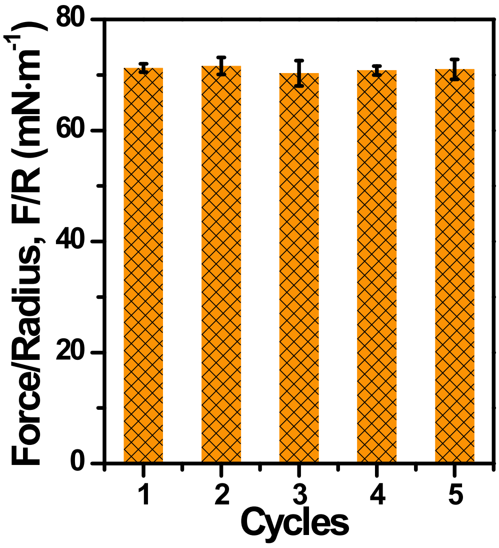

Figure S4. Adhesion of PDA during consecutive force measurements at the same interaction position.

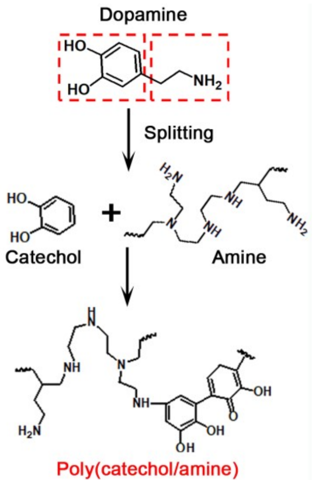

Figure S5. Schematic illustration of the merging of catechol and amine by covalent connection, which is used as a control to investigate their contribution on PDA-based adhesion.

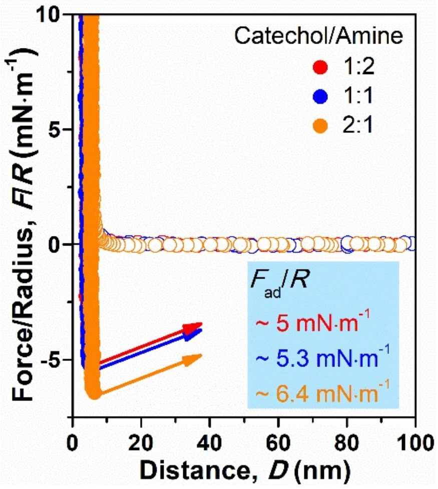

Figure S6. Representative force-distance curves of various molar ratios of amine and catechol after in situ polymerization under pH 8.5 for 1 h. Here, catechol concentration is fixed in line with dopamine (5 mM).

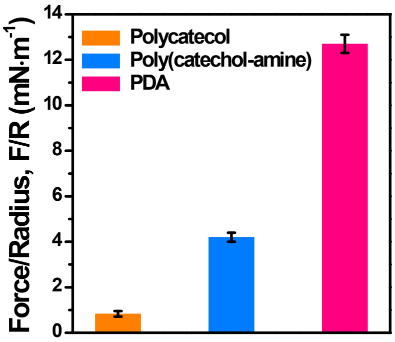

Figure S7. Adhesion between various coatings (polycatechol, poly(catechol-amine), and PDA) and mica using an asymmetric SFA measurement configuration. The results indicate that the adhesion between PDA and mica (12.7 mN·m^{-1}) is 3-fold and 15-fold higher than that of poly(catechol-amine) (4.2 mN·m^{-1}) and polycatechol (0.83 mN·m^{-1}), respectively.

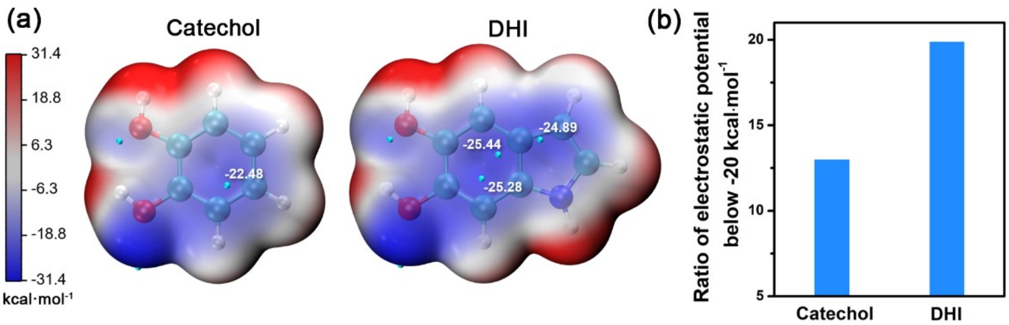

Figure S8. (a) Electrostatic potential (ESP) surfaces of DHI moieties and catechol. The ESP distribution at van der Waals surface was calculated at a M062X-D3/def2-TZVP level of theory considering the aqueous environment via SMD implicit solvent model.[19-21] (b) Ratio of electrostatic potential below -20 kcal·mol^{-1} for catechol and DHI. The result shows that DHI has higher electron densities than catechol, owing to its inherent π-conjugated structure.

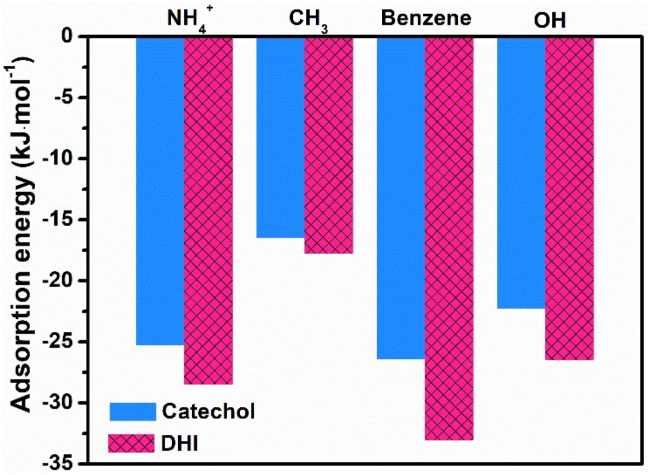

Figure S9. Adsorption energy of catechol-based moieties (catechol and DHI) and various groups (Benzene, CH_3, NH_4^+, OH). Here, the interactions between these groups and DHI/catechol represent the π-π interaction, hydrophobic interaction, cation-π interaction, and hydrogen bond, respectively. The simulation results strongly support that DHI moieties have better capacities to form these four interactions than that of catechol group.

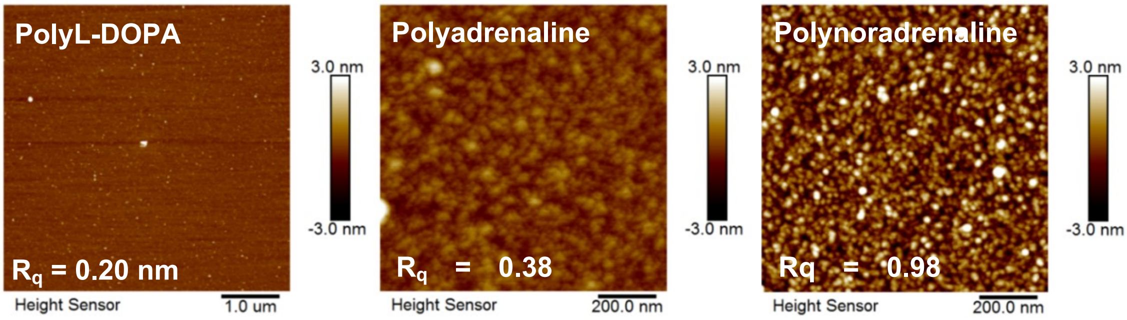

Figure S10. AFM images of polyL-DOPA, polyadrenaline and polynoradrenaline coatings after 1 h deposition

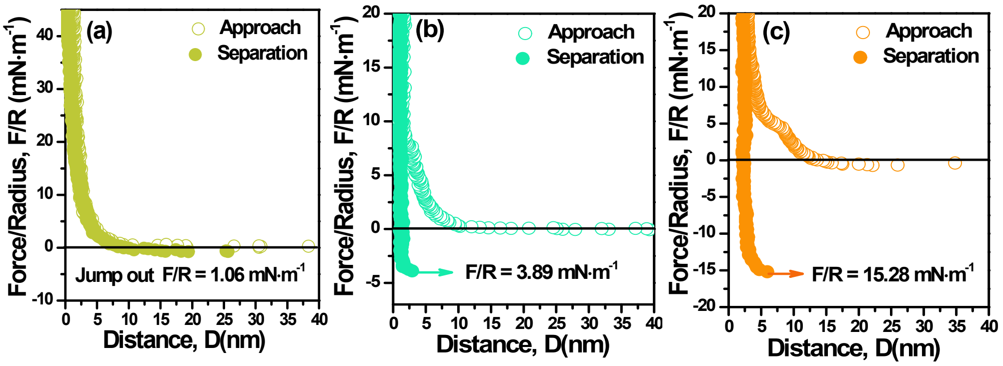

Figure S11. Representative force-distance curves between mussel-inspired derivatives coatings on mica after in situ polymerization under pH 8.5 for 1 h: (a) polyL-DOPA, (b) polyadrenaline, and (c) polynoradrenaline.

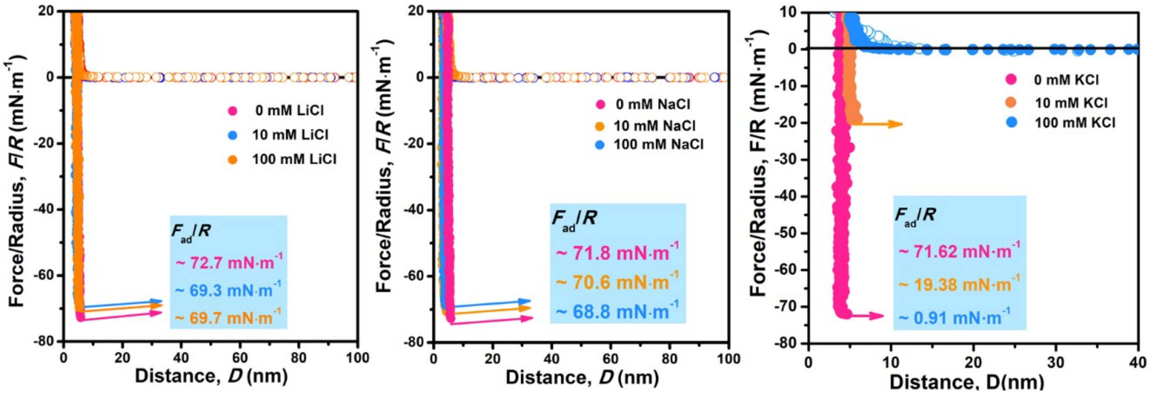

Figure S12. Representative force-distance curves of dopamine after in situ polymerization under different salt types: LiCl, NaCl, and KCl. The salt concentration is adjusted from 0 mM to 100 mM.

Table S1. Surface chemical compositions of PDA, polynoradrenaline, and polyadrenaline coatings from XPS spectra.

Sample	C1s	N1s	O1s	Si2p	Al2p
PDA	72.55	7.40	18.21	1.01	0.83
Polynoradrenaline	53.43	3.42	28.71	6.91	7.53
Polyadrenaline	8.72	1.27	53.56	21.48	14.97

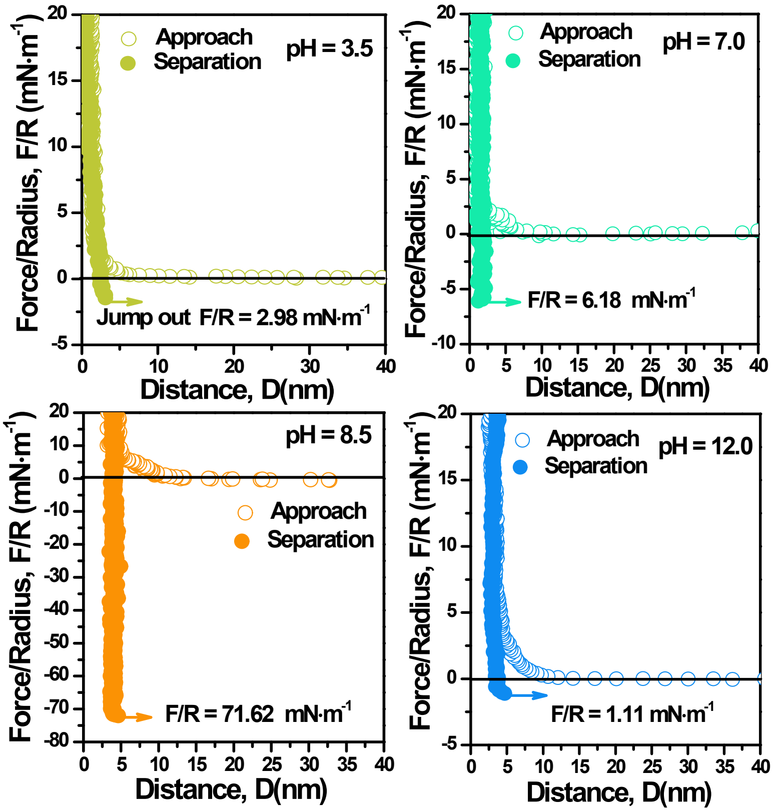

Figure S13. Representative force-distance curves between two opposing PDA coatings on mica after 1 h in situ polymerization under different pH values.

References

1. J. Israelachvili, Y. Min, M. Akbulut, A. Alig, G. Carver, W. Greene, K. Kristiansen, E. Meyer, N. Pesika, K. Rosenberg and H. Zeng, *Rep. Prog. Phys.*, 2010, **73**, 036601.
2. C. Lim, J. Huang, S. Kim, H. Lee, H. Zeng and D. S. Hwang, *Angew. Chem. Int. Ed.*, 2016, **55**, 3342-3346.
3. H. Zeng, D. S. Hwang, J. N. Israelachvili and J. H. Waite, *Proc. Natl. Acad. Sci. U. S. A.*, 2010, **107**, 12850-12853.
4. K. L. Johnson, K. Kendall, A. D. Roberts and D. Tabor, *Proc. R. Soc. London, A.*, 1971, **324**, 301-313.
5. Y. Zhao and D. G. Truhlar, *Theor. Chem. Acc.*, 2008, **120**, 215-241.
6. R. Krishnan, J. S. Binkley, R. Seeger and J. A. Pople, *J. Chem. Phys.*, 1980, **72**, 650-654.
7. S. Grimme, J. Antony, S. Ehrlich and H. Krieg, *J. Chem. Phys.*, 2010, **132**, 154104.
8. J. A. Montgomery, M. J. Frisch, J. W. Ochterski and G. A. Petersson, *J. Chem. Phys.*, 1999, **110**, 2822-2827.
9. L. Fan, L. Versluis, T. Ziegler, E. J. Baerends and W. Ravenek, *Int. J. Quantum Chem.*, 1988, **34**, 173-181.
10. C. Gonzalez and H. B. Schlegel, *J. Phys. Chem.*, 1990, **94**, 5523-5527.
11. A. V. Marenich, C. J. Cramer and D. G. Truhlar, *J. Phys. Chem. B*, 2009, **113**, 6378-6396.
12. M. J. T. Frisch, G. W.; Schlegel, H. B.; Scuseria, G. E.; Robb, M. A.; Cheeseman, J. R.; Scalmani, G.; Barone, V.; Mennucci, B.; Petersson, G. A.; Nakatsuji, H.; Caricato, M.; Li, X.; Hratchian, H. P.; Izmaylov, A. F.; Bloino, J.; Zheng, G.; Sonnenberg, J. L.; Hada, M.; Ehara, M.; Toyota, K.;

Fukuda, R.; Hasegawa, J.; Ishida, M.; Nakajima, T.; Honda, Y.; Kitao, O.; Nakai, H.; Vreven, T.; Montgomery, J. A., Jr.; Peralta, J. E.; Ogliaro, F.; Bearpark, M.; Heyd, J. J.; Brothers, E.; Kudin, K. N.; Staroverov, V. N.; Kobayashi, R.; Normand, J.; Raghavachari, K.; Rendell, A.; Burant, J. C.; Iyengar, S. S.; Tomasi, J.; Cossi, M.; Rega, N.; Millam, J. M.; Klene, M.; Knox, J. E.; Cross, J. B.; Bakken, V.; Adamo, C.; Jaramillo, J.; Gomperts, R.; Stratmann, R. E.; Yazyev, O.; Austin, A. J.; Cammi, R.; Pomelli, C.; Ochterski, J. W.; Martin, R. L.; Morokuma, K.; Zakrzewski, V. G.; Voth, G. A.; Salvador, P.; Dannenberg, J. J.; Dapprich, S.; Daniels, A. D.; Farkas, O.; Foresman, J. B.; Ortiz, J. V.; Cioslowski, J.; Fox, D. J. Gaussian 09, revision C.01; Gaussian Inc.: Wallingford, CT, 2016., *Journal*.

13. C. Y. Legault, *Université de Sherbrooke*, 2009.
14. T. Lu, *molclus program, Version 1.9.9.6, http://www.keinsci.com/research/molclus.html (accessed sep 5, 2021)*.
15. C. Bannwarth, E. Caldeweyher, S. Ehlert, A. Hansen, P. Pracht, J. Seibert, S. Spicher and S. Grimme, *WIREs Comput. Mol. Sci.*, 2021, **11**, e1493.
16. S. Grimme, C. Bannwarth and P. Shushkov, *J. Chem. Theory Comput.*, 2017, **13**, 1989-2009.
17. M. J. Frisch, J. A. Pople and J. S. Binkley, *J. Chem. Phys.*, 1984, **80**, 3265-3269.
18. S. Grimme, S. Ehrlich and L. Goerigk, *J. Comput. Chem.*, 2011, **32**, 1456-1465.
19. T. Lu and F. Chen, *J. Comput. Chem.*, 2012, **33**, 580-592.
20. J. Zhang and T. Lu, *Phys. Chem. Chem. Phys.*, 2021, **23**, 20323-20328.
21. T. Lu and F. Chen, *J. Mol. Graphics Modell.*, 2012, **38**, 314-323.
